# Advancing global health access through market shaping: Cases and learnings

**DOI:** 10.1371/journal.pgph.0004523

**Published:** 2026-02-24

**Authors:** Claire M. Wagner, Amy Lin, Sabin Nsanzimana, Janet K. Ginnard, Jayasree K. Iyer, Mary-Ann Etiebet, David Ripin, Sandeep Juneja, Dominic Hein, Gian Gandhi, Charles B. Holmes

**Affiliations:** 1 Avenir Management Partners, Boston, Massachusetts, United States of America; 2 Center for Innovation and Impact, USAID, Washington, DC, United States of America; 3 Ministry of Health, Government of Rwanda, Kigali, Rwanda; 4 Unitaid, Geneva, Switzerland; 5 Access to Medicine Foundation, Amsterdam, Netherlands; 6 Vital Strategies, New York, New York, United States of America,; 7 Clinton Health Access Initiative, Dorchester, Massachusetts, United States of America; 8 Market Access, TB Alliance, New York, New York, United States of America; 9 Vaccine Programmes & Markets, Gavi, The Vaccine Alliance, Geneva, Switzerland; 10 Global Development, Gates Foundation, Seattle, Washington, United States of America; 11 Center for Innovation in Global Health, Georgetown University, Washington District of Columbia, United States of America; London School of Economics and Political Science, UNITED KINGDOM OF GREAT BRITAIN AND NORTHERN IRELAND

## Abstract

New health products have contributed to major improvements in public health, but many clinically effective interventions still face delays in reaching low- and middle-income countries (LMICs). Market shaping approaches have emerged as a set of tools designed to address such access gaps by influencing prices, supply, and demand. Drawing on practitioner experience and illustrative cases, this paper examines how market shaping mechanisms have been used to expand access to pharmaceutical products in LMICs. We review examples including dolutegravir, rifapentine-based tuberculosis preventive therapy, pretomanid for drug-resistant tuberculosis, the RTS,S malaria vaccine, and Rwanda’s hepatitis C program, alongside ecosystem-level interventions such as revolving funds and initiatives to strengthen regional manufacturing. Across these cases, we suggest generalizable lessons and describe trade-offs related to donor dependence, supplier concentration, and timing of interventions. The paper identifies priorities for empirical research to assess the performance, risks, and applicability of market shaping tools as global health needs and resource environments evolve.

## Introduction

New health technologies can produce substantial shifts in the control of pandemics and major public health challenges. Effective polio vaccines transformed a frequently fatal or disabling infection into a disease now nearing eradication [1]. Combination antiretroviral therapy, coupled with global financing, has contributed to a nearly 70% reduction in AIDS-related deaths over the last two decades [2]. Yet new products such as these are not inevitably associated with global access [3–7].

Pharmaceutical development is complex, expensive, and unpredictable – with research and development (R&D) costs commonly estimated in the billions of dollars and development timelines exceeding a decade [8,9]. Access timelines are influenced by a range of factors, including market dynamics, availability of financing, sectoral alignment, and political commitment [10,11]. The path to access in low- and middle-income countries typically involves coordination between private sector, governments, multilateral agencies and donors—actors with distinct mandates and incentives that often diverge, complicating efforts to achieve timely and equitable delivery [12].

Against this backdrop, market shaping can be deployed to address some of these constraints. While defined in different ways [13–15], for the purposes of this article, we use the term to describe the targeted use of financial and market-based interventions to promote healthy, competitive markets and address inefficiencies that impede the timely and sustained availability of new public health tools where they are needed. A clearer understanding of market shaping instruments and ecosystem characteristics could improve practitioners’ ability to advance product access, particularly amid shifting donor priorities, emerging fiscal constraints, and increasing attention on technology-enabled innovation and regional manufacturing [16,17].

In light of these dynamics, this paper offers a practitioner perspective on the use of market shaping in global health, drawing on direct experience with related institutions and initiatives and on a session held at the 2023 Consortium of Universities in Global Health (CUGH) conference [18]. Rather than a systematic review, it uses illustrative cases to examine the evolving role of market shaping mechanisms in expanding access to health products, identify common trade-offs, and highlight questions that merit further empirical study within a changing global health architecture.

### Approaches to market shaping and market shaping tools in today’s global health ecosystem

The expansion of market shaping activities in global health occurred alongside the substantial rise in development assistance for health (DAH) from 2000-2020. Between 2000–2015, DAH for HIV/AIDS, malaria, tuberculosis, maternal health, and newborn and child health grew by an average of 10% annually – totaling approximately $255 billion [19] – although growth has slowed in recent years. During most of this period, the U.S. government (via USAID and other agencies) was a leading global health donor; however, recent reductions and policy shifts have altered this landscape [20]. Consistent with historic DAH funding priorities, most market shaping efforts have focused on infectious diseases, with comparatively fewer examples targeting non-communicable disease products.

Global health institutions, supported by increases in DAH, have played central roles in advancing access to essential health technologies through market shaping. These organizations have used a range of strategies and frameworks to address market inefficiencies, promote affordability, and stimulate sustainable supply (Table 1).

**Table 1:** Examples of Market Shaping Strategies by Institution.

Institution	Market Shaping Strategy
USAID* [21]	Three-pronged approach addressing market inefficiencies by reducing transaction costs, improving market information, balancing supplier and buyer risks.
**Global Fund** [22]	NextGen Market Shaping Framework that promotes collaborative procurement and equitable access to quality-assured TB, HIV, and malaria products.
**Gavi** [23,24]	Market Shaping Strategy 5.0 (2021–2025) emphasizing sustainable and competitive supply, predictable demand, enabling conditions for innovation.
**Unitaid** [25]	Market-based model that identifies and addresses access barriers to ensure products are fit-for-purpose, quality-assured, and affordable, using a range of market shaping approaches to facilitate product introduction and equitable access at scale.
**Clinton Health Access Initiative (CHAI)** [26]	Implements cross-cutting market shaping activities spanning procurement, financing, and innovation to enhance access.
**MedAccess** [27]	Provides financial guarantees and risk-sharing mechanisms to lower prices, expand supply, and stabilize markets for priority health products.

* *Since the time of original manuscript submission (2024), USAID was merged into the U.S. State Department and its future role in global health market shaping continues to evolve.*

While market shaping extends beyond procurement, pooled procurement mechanisms*—*such as those coordinated by Gavi, UNICEF, and the Global Fund*—*have contributed to greater availability of key health commodities [28]. These mechanisms depend on an expectation of funding and manufacturer confidence that procurement arrangements will be sustained [29]. Risk-sharing instruments provided by organizations such as the Gates Foundation and MedAccess have supported this by mitigating supplier uncertainty for priority products. Market shaping initiatives aim to improve access through coordinated demand and efficient purchasing – approaches that, as demonstrated in the PEPFAR antiretroviral program, can generate savings to reinvest into expanded health service delivery [30]. Broader activities that promote market access encompass interrelated domains including regulatory alignment, global and domestic policymaking, distribution strategies, community engagement, and post-introduction scale-up [31].

### Examples of market shaping programs

By the time a healthcare product successfully reaches patients in LMICs, numerous transactions have occurred among manufacturers, funders, distributors, procurement agencies, and public administrators. Some of these transactions attempt to correct market imperfections through risk-sharing or risk-shifting arrangements between suppliers and purchasers. Establishing such mechanisms requires functional capabilities across business development, legal, and strategic financing domains.

The following section presents five illustrative cases of market shaping efforts for specific products, followed by examples that influence the broader market shaping ecosystem. These cases were selected to represent interventions addressing “demand health,” “supply dynamics,” and the alignment of innovations with country needs and “clear pathways” to uptake, adapted from Gavi’s Health Markets Framework. They include both long-standing and emerging global health challenges, and span from small molecule therapeutics to vaccines. The selection is not exhaustive; other literature documents additional approaches and variable outcomes [32–35]. Across these cases, we aim to elucidate mechanisms that facilitated access and the trade-offs and uncertainties that accompany them. For broader overviews of interventions, we refer readers toward the 2023 Report by Rethink Priorities [36], a 2023 Insight piece by MedAccess [37], the 2014 Market Shaping Primer by USAID’s Center for Innovation and Impact [38], and a recent systematic review [39].

### Market shaping cases

#### Case 1: Dolutegravir.

The field of HIV treatment has evolved to include less toxic and more effective antiretroviral drugs that are simpler for individuals to take than prior regimens. The integrase inhibitor, dolutegravir (DTG), is one such drug that rapidly suppresses viral load and has a favorable side effect profile compared to earlier HIV drugs. Yet in 2014, a year after its approval by the U.S. FDA, price and volumes posed challenges for global use [40]. In response, DTG’s originator company, ViiV Healthcare targeted supply health by granting a direct license to Aurobindo Pharma, and signed an agreement with the Medicines Patent Pool to allow for voluntary patent licenses for the production of generic pediatric and adult formulations of the drug [41]. The follow-on challenge was to ensure prices were low enough to promote access but also sufficiently attractive to engage potential manufacturers—a balance that was reached in September 2017.

That year, a collaborative effort between CHAI, Gates Foundation, UNAIDS, Unitaid, PEPFAR, the Global Fund, civil society, and community partners, led to several outcomes: (1) a ceiling price of approximately $75 per person per year; and (2) an advance purchase agreement (APA) between USAID and two generic manufacturers; [42,43] and (3) funding by Unitaid and others to support civil society and community organizations to accelerate adoption. The ceiling price was designed to enable access, the APA targeted “demand health,” and community engagement served to support “clear pathways” for country uptake for this innovation. The APA enabled the two generic suppliers to start production earlier, instead of waiting for individual country orders to emerge through traditional tendered processes. HIV advocates called for access, further highlighting the demand to hasten access to DTG in LMICs.

By July 2019, nearly 3.9 million people living with HIV across 61 countries had access to generic DTG or a fixed-dose combination containing DTG, representing 9.3% of the total global population living with HIV at the time. In 2020, ViiV received FDA approval for a pediatric dispersible formulation of DTG, and through a separate market shaping partnership, a generic formulation made by Viatris received US FDA tentative approval through PEPFAR five months later [44]. The collaboration between Unitaid, CHAI, originator ViiV Healthcare, and generic manufacturers Viatris and Macleods enabled this accelerated pathway, with the generic pediatric formulation receiving tentative regulatory approval within months – an unusually short timeline compared to multi-year delays that historically separate originator and generic ARV products [45]. As a result of these partnerships, additional country registrations by generic manufacturers, and procurement supported by Unitaid and CHAI in 13 high-volume markets, by 2021 more than 22 million people in 110 LMICs were receiving DTG, at prices below $40 per patient per year [46,47].

Although the DTG case demonstrates that coordination, risk-sharing, and community-engagement can accelerate access, it also highlights dependencies with implications for long-term sustainability. APAs in particular – while enabling risk-sharing – can have consequences including overbuying and overpaying, and their extensive use by high-income countries during COVID-19 contributed to inequitable access in LMICs [48]. Such multi-party agreements also rely on donor financing and cross-institutional coordination, both vulnerable to shifts in funding priorities. Early market concentration among a small number of licensed manufacturers may also narrow the competitive landscape and increase exposure to supply or price volatility over time. While the DTG partnership established a new benchmark for affordability and reach, it raises questions on how market shaping models—particularly in less well financed disease areas—can maintain competitive dynamics, operational flexibility, sustainability, and country-led priority-setting and stewardship as donor involvement declines.

#### Case 2: Tuberculosis preventive treatment and therapeutics.

Clinical studies over the last decade have demonstrated that 3-month and 1-month isoniazid and rifapentine-based regimens (3HP and 1HP respectively) were noninferior to a 9-month isoniazid only regimen for tuberculosis (TB) preventive therapy and achieve higher completion rates [49–51]. In 2018, rifapentine – a drug first approved for use in 1998 – was only available through the originator company, Sanofi. Partners, including Unitaid, the Global Fund, and the Stop TB Partnership (through the Global Drug Facility) collaborated with Sanofi in 2019 to establish an access price for 150mg rifapentine tablets. In 2021, Unitaid and CHAI established a product development incentive program targeting both “demand health” and “supply dynamics” to commercialize the first fixed-dosed combination of 3HP through the Indian manufacturer, Macleods Pharmaceutical Limited [52]. A subsequent demand-side intervention included a volume guarantee to extend the access pricing and expand Macleods’ 3HP production capacity [53]. A third manufacturer, Lupin, began producing 3HP in 2022 concurrent with price agreements that established a single ceiling price ($14.25 USD) in 138 LMICs [54]. These agreements reduced prices by 21–32%, depending on the regimen [55,56].

Market shaping tools were also applied to expand use of the TB Alliance’s pretomanid, a component of shortened, all-oral WHO-recommended treatment regimens for drug-resistant TB (DR-TB) called BPaL/M, adopted as standard of care for DR-TB since 2020 [57]. The initial WHO recommendation for pretomanid, however, was limited to pre-extensively drug resistant TB (approximately 12,000 treatments annually) and constrained market size. Working with the TB Alliance, the commercial manufacturer, Viatris, agreed to provide the drug through Stop TB Partnership’s Global Drug Facility at an access price of $364 per course for 150 countries and territories [58]. Fifteen high-burden early adopters initiated demonstration projects, and by May 2023, 109 countries were using all-oral, six-month BPaL-based regimens [59].

Concurrently, TB Alliance and Médecins Sans Frontières (MSF) generated additional data that supported the WHO’s 2022 expansion of eligibility to all people with multi-drug resistant TB. The TB Alliance then worked with 13 high burden countries to project 5-year demand for pretomanid, forming the basis for a “demand health” volume guarantee partnership between TB Alliance, Viatris, and MedAccess. This intervention lowered the price for eligible countries by 34% to $240 per course following the WHO guidelines update [60].

Within a year of the revised guidance, more than 70 countries had purchased over 40,000 courses of pretomanid, representing a market share of 25–30% of the potential multi-drug resistant TB market. Under license from the TB Alliance, additional manufacturers, including Macleods and Remington, have since obtained WHO pre-qualification, potentially further expanding access [61].

While the 3HP and pretomanid experiences show that targeted incentives and coordinated financing can expand access to both preventive and curative TB therapies, they also illustrate the complexity of deploying multiple market shaping instruments simultaneously. Volume guarantees, price ceilings and donor-backed purchasing commitments have been associated with scale-up, but their concurrent application can reduce the clarity of market signals, introduce uncertainty into manufacturers’ long-term planning, and increase reliance on donors. In settings without sustained procurement or diversified demand, suppliers may face challenges maintaining production at access price points, with possible implications for innovation and the incorporation of future product improvements. Further research could clarify how these mechanisms influence competition and long-term sector viability in global health markets, including their implications for equity and country ownership [62].

#### Case 3: Malaria vaccine.

First-in-class product introductions present additional challenges related to uncertain policy landscapes, regulatory pathways, and demand forecasts. The case of the RTS,S malaria vaccine, which targets the prevention of clinical malaria due to *P. falciparum* in children, illustrates these challenges. While other literature details the broader development and rollout of RTS,S [63–65] this case highlights a single market shaping intervention designed to mitigate supply risk during policy deliberation.

Before issuing its first Malaria Vaccine Guidelines [66], WHO’s Strategic Advisory Group of Experts on Immunization (SAGE) and the Malaria Policy Advisory Group (MPAG) required a demonstration project called the Malaria Vaccine Implementation Programme to assess feasibility and public health impact. Readouts from this program were pending when Gavi considered whether to include it in its Gavi’s Vaccine Investment Strategy [67].

To avoid production delays while awaiting these normative guidelines, a “supply dynamics” market intervention was deployed to de-risk the manufacturer [68]. Gavi committed to fund the originator, GlaxoSmithKline (GSK), to maintain full-scale production of the RTS,S antigen for up to three years, with the provision that Gavi would later receive a credit of equal value toward future procurement if the vaccine program were approved. MedAccess insured Gavi against financial loss in the event that the malaria vaccine was not approved by SAGE and MPAG. This arrangement bridged a period of acute uncertainty, enabling uninterrupted production and higher initial launch volume in 2023 than would otherwise have been feasible [69].

However, this form of pre-approval risk-sharing also highlights a structural trade-off in market shaping: the balance between early preparedness and fiscal conservatism. Such de-risking instruments have opportunity costs, particularly in contexts characterized by uncertain demand certainty or pending policy endorsement. Future analyses could assess how these mechanisms affect patterns of public sector reliance, private sector expectations, and resource allocation dynamics within global health portfolios.

#### Case 4: HCV treatment.

Sofosbuvir-Ledipasvir, a once-daily oral antiviral, was introduced in the U.S. market in 2014 at a cost of about $90,000 per patient per cure; itself a multiple of GDP per capita of many LMICs. At that time, access to diagnostics and treatment for hepatitis C virus (HCV) was limited in most LMICs. Available treatments were interferon-based, costing over $10,000 per patient with modest cure rates (50%) and substantial side effects. At the same time, programmatic data from Unitaid and Médecins Sans Frontières demonstrated that simplified, decentralized care models for hepatitis C could achieve high cure rates in low-resource settings [70,71].

Seeking better options, Rwanda’s Ministry of Health partnered with Gilead, an originator for direct-acting antivirals for HCV treatment, to introduce Sofosbuvir-Ledipasvir at $1,200 for a 3-month course [72]. A 2016 screening program for high-risk populations – including people living with HIV – quantified demand and informed advocacy for Global Fund procurement support. This contributed to a further price reduction to $780 USD per course, alongside subsidized access to antibody rapid tests and confirmatory viral load diagnostics for HIV-HCV co-infected patients.

Rwanda became the first African country to implement an HCV elimination plan in 2018, targeting screening for 7 million people aged 15 and above and treatment for all who tested positive [44]. Following the plan’s launch, Rwanda negotiated additional price reductions, including $0.75 per rapid test and a 60% reduction in the cost of viral load cartridges for both HIV and HCV.

Recognizing the role treatment plays in the uptake of screening, the Rwandan government further negotiated a $60 per person for the full three-month treatment course—the lowest documented price for WHO Pre-Qualified Direct-Acting Antivirals. Rwanda’s interventions illustrate mechanisms that target “demand health” and “clear pathways” for country uptake. As of January 2024, approximately 7 million Rwandans have been screened and 60,000 have been treated, with national prevalence estimated to be 1%, aligning the country with WHO elimination targets for 2030 [73].

Rwanda’s experience demonstrates how nationally driven and coordinated public sector action, data-informed negotiation, and donor collaboration can expand access to diagnostics and therapeutics. Yet its success also introduces considerations regarding replicability and long-term market viability. Price reductions, while improving affordability, may be challenging to sustain in settings that have limited demand visibility, constrained procurement capacity or insufficient diagnostic infrastructure. More broadly, the compression of margins may affect manufacturer incentives to maintain supply or invest in developing related products. As additional countries adopt similar approaches, further analysis could clarify how public sector-led market shaping can support access while maintaining private sector engagement and innovation over time.

#### Case 5: Ebola vaccine.

Unpredictable demand characterizes many products intended for emerging pathogen prevention and control [74]. Because Ebola causes infrequent and, historically, small outbreaks, commercial incentives to develop a vaccine or maintain a diverse or competitive market are minimal. In such contexts, market mechanisms have included “push” funding to invest in early stage development and fully funded stockpiles to maintain production for deployment as needed [75].

The world’s first Ebola vaccine—a VZV-based live attenuated vaccine initially engineered by scientists from the Public Health Agency of Canada’s National Microbiology Laboratory was licensed in 2014 to Merck for further development. Following its use under “expanded access” in Guinea in 2015 and the Democratic Republic of Congo (2018–2020), the vaccine was licensed as ERVEBO (Ebola Zaire Vaccine, Live) in 2019 by the European Medicines Agency and prequalified by WHO [76].

Subsequent partnerships helped ensure broader availability in future outbreaks. Merck, in collaboration with UNICEF, WHO, and Gavi established the world’s first global Ebola vaccine stockpile with ERVEBO in 2021. The stockpile, which now has approximately 500,000 doses, is governed by the International Coordinating Group on Vaccine Provision and operates under guidance from the WHO SAGE on immunization [77]. Merck supplies the vaccine at cost, while Gavi finances the stockpile and deployment for at-risk populations in Gavi-supported countries, improving “demand health” and readiness for rapid response [78,79].

This model, while designed to support preparedness, also reflects a structural trade-off between readiness and long-term sustainability. Maintaining production capacity, warehousing, and stockpile for a vaccine that may remain unused for extended periods requires stable financing and institutional commitment—conditions that can be challenging to sustain amid shifting global health priorities. Although examples of long-term at-cost supply arrangements exist – such as Merck’s Mectizan Donation Program which has been running for nearly four decades [80] – they inherently reduce seller profitability, raising questions regarding sustainability of private sector participation. These factors underscore the challenge of designing market shaping mechanisms that remain viable under conditions of uncertain demand.

### Enhancing broader ecosystems for medicines access

Access has also been advanced through broader, product agnostic ecosystem changes. These include the addition of new institutions and funds, strategic shifts within existing ones, and normative mechanisms that support competition.

The Medicines Patent Pool (MPP), founded in 2010 and funded primarily by Unitaid was established to address IP issues barriers that delay access. MPP negotiates voluntary licenses, pools patents to enable generic manufacturing, and supports development of new formulations in collaboration with public and private sector partners. This approach has enabled supplier diversification and access for products across a portfolio of diseases and conditions [81].

UNICEF’s Vaccine Independence Initiative (VII), launched in 1991, operates as a revolving fund to support countries as they use and strengthen their public procurement mechanisms for health-related products. From January 2020 through September 2024, VII-enabled transactions accelerated access – typically by 2–4 months – of approximately $1.7B USD in health supplies including nearly 1B doses of vaccines and 9M cartons of ready-to-use therapeutic food. Since its inception, the mechanism has supported more than 100 countries in product procurement [82]. It has also been used for pre-financing of COVID vaccines and in 2023 enabled greater than $200M USD of health products across 32 countries [83]. Similarly, the Global Fund established a revolving fund facility in 2023 backed by a $100 million Gates Foundation investment. Its early transactions have focused on improving national uptake of mosquito nets through volume guarantees for sellers and price reductions for buyers [84]. If aligned with country need, these facilities have the potential to accelerate the path to market for new technologies, particularly in fragmented markets.

In response to vaccine inequities exposed during the COVID-19 pandemic, the Africa Centres for Disease Control and Prevention (Africa CDC) established the Partnership for African Vaccine Manufacturing initiative to increase the share of vaccines produced on the continent from less than 1% today to 60% by 2040 [85]. This initiative has since broadened to include other health products. Its progress depends multiple factors including technology transfer, a skilled manufacturing workforce, and predictable purchasing agreements [86].

Complementing this effort, Gavi introduced the African Vaccine Manufacturing Accelerator in 2024, capitalized with US$ 1.2 billion. The mechanism provides milestone payment to vaccine manufacturers in Africa that achieve WHO Prequalification of vaccines suitable for Gavi-supported programs; and a per-delivered-dose “top-up” basis to African manufacturers that secure UNICEF procurement contracts. This model aims to improve the commercial viability of regional vaccine production.

The Access to Medicine Foundation, established in 2003, promotes transparency and accountability in the pharmaceutical industry. Its Access to Medicine Index benchmarks company performance on access-related metrics, including pricing, licensing, and equitable R&D practices. By publishing comparative assessments, the Foundation seeks to encourage private-sector engagement in access strategies.

## Discussion

Market shaping interventions have become operational tools to expand access to essential health products in LMICs. Across the cases presented – including antiretroviral therapy, tuberculosis drugs, malaria vaccines, and outbreak-response countermeasures – coordinated efforts to influence supply, pricing and demand have been associated with increased uptake, lower prices and reduced supply risks. These outcomes demonstrate the effects of market shaping while also revealing trade-offs and uncertainties that merit further examination, particularly as countries and donors consider incorporating such approaches into long-term health system strategies.

A recurring question across the examples is the potential trade-off between global coordination and local adaptability. Centralized price negotiations and pooled procurement have yielded affordability gains, but may reduce flexibility for responses tailored to local epidemiology. In cases such as DTG and 3HP, early consolidation of purchasing power enabled rapid introduction yet relied mainly on global market intelligence rather than facility-level data. Empirical evidence around these trade-offs remains limited, partly because market shaping interventions are not conducive to controlled evaluations. The Affordable Medicines Facility-malaria (AMFm) does, however, offer insights: a large-scale subsidy program reduced consumer prices and increased uptake of quality-assured artemisinin-based combination therapies. Yet its impact varied across countries, private-sector engagement was uneven, and sustainability became uncertain after subsidy adjustments [87].

The presented cases also illustrate that market shaping occurs at different points in the product lifecycle. Most examples—dolutegravir, 3HP, pretomanid, and Rwanda’s hepatitis C agreement—involved post-approval mechanisms such as licensing, coordinated pricing, and volume guarantees implemented after regulatory paths were defined. By contrast, the RTS,S vaccine involved pre-approval shaping, with Gavi and MedAccess supporting antigen production during a period of policy uncertainty to avoid supply delays after a pending WHO recommendation. A related example of early alignment is PNEUMOSIL, a 10-valent PCV vaccine co-developed by PATH and the Serum Institute of India, Pvt. Ltd. with Gates Foundation funding, where coordination of product design, financing, and anticipated demand, supported lower pricing and local production [88]. Whether this end-to-end model is generalizable across contexts merits further study.

Considerations related to resilience also emerge across mechanisms. Advance purchase commitments, long-term agreements, and volume guarantees can mitigate production risk and support manufacturing scale-up, but often engage a narrow set of suppliers. The DTG and 3HP examples show how supplier concentration can facilitate access but could result in vulnerability if those suppliers face disruptions or withdraw due to thin margins. These dynamics raise questions about supplier diversity and the development of regional and local pharmaceutical manufacturing capacity. Significant price reductions may make it difficult for regional manufacturers to compete. Conversely, mechanisms such as those used by MPP may support the participation of local or regional producers, including when early alignment – such as in the PNEUMOSIL case – lays the foundation for lower prices at scale. Comparative evidence on how market shaping tools affect long-term viability of local infrastructure remains sparse.

Several governments are beginning to institutionalize market shaping functions within national procurement and supply chain agencies. For example, Ethiopia’s Pharmaceutical Supply Agency has created a Quantification and Market Shaping Directorate to analyze market dynamics, diversify suppliers, and manage a revolving drug fund [89,90]. Such internal models offer promise for shortening feedback loops between health authorities, procurement units and manufacturers, and may enable more adaptive, country-led, context-specific decision-making than global mechanisms alone. Comparative studies could help clarify the relative effectiveness of national, regional and global market shaping approaches.

A central issue concerns whether market shaping tools can transition from donor-led initiatives to durable systems functions. Many large-scale interventions continue to rely on predictable multilateral donor contributions, which are becoming less certain. The ability of emerging financing approaches, such as Gavi’s collaborations with development finance organizations to support capital-intensive, multi-year market interventions merits further study [91]. As these models evolve, empirical assessment will be needed to understand how different forms of capital affect market stability, supplier incentives, and countries’ planning capacity.

Potential risks also warrant examination. Price ceilings and pooled procurement can reduce costs but may compress manufacturer margins or affect incentives for continued product development [92]. Some also suggest pooled procurement may delay transitions to newer products better aligned with national needs [93]. As regional manufacturers assume a larger role, their financial viability will be shaped by policy decisions and regional and domestic pricing. Experiences from institutions such as the Institut Pasteur de Dakar and Instituto Butantan indicate that sustained public-sector support has been important for maintaining local production capacity.

Regulatory and quality assurance processes, although outside this article’s scope, remain central to access and outcomes. Regional harmonization efforts – such as the African Medicines Agency and comparable frameworks in Southeast Asia and Latin America – reflect attempts to improve efficiency and reduce barriers for manufacturers seeking to supply multiple LMIC markets [94–96]. These mechanisms have potential to realize efficiencies and improve access to quality-assured products.

Looking ahead, as global health priorities expand to include non-communicable diseases, an open question is the extent to which current market shaping approaches can be adapted to therapeutic areas with diffuse markets, less well-defined delivery models, or chronic treatment courses. Early efforts, such as programs led by BIO Ventures for Global Health, suggest some principles may be adaptable, but broader evidence remains limited.

Finally, effective market interventions depend on a shared understanding of constraints, informed by robust market intelligence and clarity on intervention goals, risks, and design. As global health financing becomes more limited, and the institutional landscape evolves, coordinated approaches may become more difficult to implement, potentially requiring participating organizations to tolerate greater levels of risk.

## Conclusion

Market shaping interventions will continue to evolve at the intersection of product-specific deals, broader ecosystem trends, and new forms of partnership. The cases presented here illustrate how targeted mechanisms can support uptake, reduce uncertainty and distribute risk. They also each underscore that outcomes depend on context – shaped by the reliability of financing, funder prioritization, the incentives facing private sector manufacturers, and institutional capabilities of public sector agencies. As these tools continue to evolve, their effectiveness may also depend on how well  existing globally coordinated approaches can align with and work in support of the increasing emphasis on country- and region-defined supply security, industrial policies, and procurement mechanisms.

As the global health funding landscape shifts, empirical work will be needed to characterize the performance of different market shaping tools, understand their individual and combined effects, and assess their potential risks and unintended consequences. Changing disease burdens in LMICs will also require a clearer understanding of which approaches are most effective in expanding access across emerging therapeutic areas. Taken together, the interventions described highlight the value of examining distinct cases while distilling broader lessons for partnership design, financing strategies, and measurable, long-term health system outcomes.

## References

[1] Thompson KM. Polio eradication: what kind of world do we want?. Lancet Infect Dis. 2022;22(2):161–3. doi: 10.1016/S1473-3099(21)00458-8 34648732 PMC8504921

[2] UNAIDS. The path that ends AIDS. 2023. Available at: https://thepath.unaids.org/wp-content/themes/unaids2023/assets/files/2023_report.pdf

[3] Mao W, Hodges EU, Zimmerman A, Ortiz EJ, Kilburn K, Silimperi D, et al. Development, launch, and scale-up of health products in low-income and middle-income countries: a retrospective analysis on 59 health products. Lancet Glob Health. 2025;13(6):e1132–9. doi: 10.1016/S2214-109X(25)00062-2 40412401 PMC12100461

[4] Ong M. A comprehensive framework identifying barriers to global health R&D innovation and access. BMJ Glob Health. 2023;8(9):e013076. doi: 10.1136/bmjgh-2023-013076 37751936 PMC10533694

[5] Ravinetto R, Henriquez R, Srinivas PN, Bradley H, Coetzee R, Ochoa TJ, et al. Shaping the future of global access to safe, effective, appropriate and quality health products. BMJ Glob Health. 2024;9(1):e014425. doi: 10.1136/bmjgh-2023-014425 38195155 PMC10807033

[6] Wouters OJ, Kuha J. Low- And Middle-Income Countries Experienced Delays Accessing New Essential Medicines, 1982-2024. Health Aff (Millwood). 2024;43(10):1410–9. doi: 10.1377/hlthaff.2024.00089 39374462

[7] Cockburn IM, Lanjouw JO, Schankerman M. Patents and the Global Diffusion of New Drugs. Am Econ Rev. 2016;106(1):136–64. doi: 10.1257/aer.20141482 29546973

[8] Schlander M, Hernandez-Villafuerte K, Cheng C-Y, Mestre-Ferrandiz J, Baumann M. How Much Does It Cost to Research and Develop a New Drug? A Systematic Review and Assessment. Pharmacoeconomics. 2021;39(11):1243–69. doi: 10.1007/s40273-021-01065-y 34368939 PMC8516790

[9] Wouters OJ, McKee M, Luyten J. Estimated Research and Development Investment Needed to Bring a New Medicine to Market, 2009-2018. JAMA. 2020;323(9):844–53. doi: 10.1001/jama.2020.1166 32125404 PMC7054832

[10] Bigdeli M, Jacobs B, Tomson G, Laing R, Ghaffar A, Dujardin B, et al. Access to medicines from a health system perspective. Health Policy Plan. 2013;28(7):692–704. doi: 10.1093/heapol/czs108 23174879 PMC3794462

[11] Wirtz VJ, Hogerzeil HV, Gray AL, Bigdeli M, de Joncheere CP, Ewen MA, et al. Essential medicines for universal health coverage. Lancet. 2017;389(10067):403–76. doi: 10.1016/S0140-6736(16)31599-9 27832874 PMC7159295

[12] Moon S, Bermudez J, ’t Hoen E. Innovation and access to medicines for neglected populations: could a treaty address a broken pharmaceutical R&D system?. PLoS Med. 2012;9(5):e1001218. doi: 10.1371/journal.pmed.1001218 22615544 PMC3352855

[13] World Health Organization. Market shaping for access to medical products: a practical guide. Geneva: World Health Organization. 2021.

[14] Gavi, the Vaccine Alliance. Gavi market shaping approach. Geneva: Gavi, the Vaccine Alliance. 2018.

[15] Clinton Health Access Initiative. Market shaping primer. Boston, MA: Clinton Health Access Initiative. 2020.

[16] Jooste I. No one can replace the U.S. as it retreats from global health. Science. 2025;388(6743):138–9. doi: 10.1126/science.ady0874 40209000

[17] Gupta R. The need for global access to biomedical innovations during pandemics. Nat Biotechnol. 2021;39(6):664–6. doi: 10.1038/s41587-021-00942-3 34031585

[18] Consortium of Universities for Global Health. Market shaping and global health: Accelerating access to novel pharmaceutical products worldwide. Available at: https://static1.squarespace.com/static/6298f70d5e12f455136a80a7/t/6436bb05b645b20d4acb742a/1681308422639/FINAL+Conference+Program+Agenda.pdf#h.s53k4qsi13wi

[19] Dieleman JL, Schneider MT, Haakenstad A, Singh L, Sadat N, Birger M, et al. Development assistance for health: past trends, associations, and the future of international financial flows for health. Lancet. 2016;387(10037):2536–44. doi: 10.1016/S0140-6736(16)30168-4 27086170

[20] Clark RA, McQuaid CF, Richards AS, Bakker R, Sumner T, Prŷs-Jones TO, et al. The potential impact of reductions in international donor funding on tuberculosis in low-income and middle-income countries: a modelling study. Lancet Glob Health. 2025;13(9):e1517–24. doi: 10.1016/S2214-109X(25)00232-3 40659023

[21] USAID Center for Innovation and Impact. Healthy Markets for Global Health: A Market Shaping Primer. Available at: https://www.usaid.gov/cii/market-shaping-primer

[22] Market Shaping Strategy. 34th Board Meeting Market Shaping Strategy. 2015, November 15. https://archive.theglobalfund.org/media/4200/archive_bm34-17-annex1marketshapingstrategy_paper_en.pdf

[23] Gavi. Market Shaping 2024. Available at: https://www.gavi.org/our-alliance/market shaping

[24] Gavi Alliance Market Shaping Strategy 2021-2025. Available at: https://www.gavi.org/sites/default/files/about/Gavi-Alliance-Market shaping-Strategy-2021-2025.pdf

[25] Untaid. Unitaid Strategy 2023-2027. Available at: https://unitaid.org/assets/Unitaid_Strategy_2023-2027.pdf

[26] CHAI. CHAI Market Shaping Framework. 2024. https://www.clintonhealthaccess.org/wp-content/uploads/2024/02/CHAI-Market%20shaping-Framework-Feb2024.pdf

[27] MedAccess. Our Innovative Finance Products. Available at: https://medaccess.org/innovative-finance/our-innovative-finance-products/

[28] Gehl Sampath, Padmashree, Market Shaping and Market Access in the Global Vaccines Market: Approaches for the Future (October 02, 2021). Available at SSRN: https://ssrn.com/abstract=3945668

[29] Yadav P. Health Product Supply Chains in Developing Countries: Diagnosis of the Root Causes of Underperformance and an Agenda for Reform. Health Syst Reform. 2015;1(2):142–54. doi: 10.4161/23288604.2014.968005 31546312

[30] Holmes CB, Coggin W, Jamieson D, Mihm H, Granich R, Savio P, et al. Use of generic antiretroviral agents and cost savings in PEPFAR treatment programs. JAMA. 2010;304(3):313–20. doi: 10.1001/jama.2010.993 20639565

[31] Moon S, Chowdhury S, Schäferhoff M, et al. Analyzing global health access architecture: pathways for equitable product availability. BMJ Glob Health. 2022;7(3):e008340. doi: 10.1136/bmjgh-2021-008340

[32] Cavaller Bellaubi M, Harvey Allchurch M, Lagalice C, Saint-Raymond A. The European Medicines Agency facilitates access to medicines in low- and middle-income countries. Expert Rev Clin Pharmacol. 2020;13(3):321–5. doi: 10.1080/17512433.2020.1724782 32053756

[33] Malhotra S, Cameron A-I, Gotham D, Burrone E, Gardner PJ, Loynachan C, et al. Novel approaches to enable equitable access to monoclonal antibodies in low- and middle-income countries. PLOS Glob Public Health. 2024;4(7):e0003418. doi: 10.1371/journal.pgph.0003418 38950021 PMC11216602

[34] Dellepiane N, Pagliusi S, Registration Experts Working Group. Challenges for the registration of vaccines in emerging countries: Differences in dossier requirements, application and evaluation processes. Vaccine. 2018;36(24):3389–96. doi: 10.1016/j.vaccine.2018.03.049 29724510 PMC6278877

[35] McCarthy K, Ramarao S, Taboada H. New dialogue for the way forward in maternal health: addressing market inefficiencies. Matern Child Health J. 2015;19(6):1173–8. doi: 10.1007/s10995-014-1636-3 25467178

[36] Kudymowa J, Hu J, Basnak M. An overview of market shaping for global health: landscape, new developments, and gaps. Rethink Priorities. September 2023. Accessed March 18, 2025. https://rethinkpriorities.org/wp-content/uploads/2023/09/An-overview-of-marketshaping-in-global-health-Landscape-new-developments-and-gaps.pdf

[37] Teo M. How do advance market commitments and volume guarantees shape healthcare markets? MedAccess. November 30, 2023. Accessed March 18, 2025. https://medaccess.org/how-do-advance-market-commitments-and-volume-guarantees-shape-healthcare-markets/

[38] Center for Accelerating Innovation and Impact. 2014. Healthy markets for global health: A market shaping primer. United States Agency for International Development. https://perma.cc/M4RD-KTC4

[39] Mao W, Olson K, Urli Hodges E, Udayakumar K. Progress, Impacts and Lessons from Market Shaping in the Past Decade: A Systematic Review. Available at SSRN: https://ssrn.com/abstract=4726177 or http://dx.doi.org/10.2139/ssrn.4726177 10.3389/fpubh.2025.1614471 PMC12408518 40917430

[40] Sim J, Hill A. Is pricing of dolutegravir equitable? A comparative analysis of price and country income level in 52 countries. J Virus Erad. 2018;4(4):230–7. 30515303 10.1016/S2055-6640(20)30311-3 PMC6248837

[41] Special Edition Report Dolutegravir. Initiative for Medicine Access & Knowledge. Available at: http://www.i-mak.org/wp-content/uploads/2017/06/I-MAKRoadmapSEReportDTG20170619F.pdf

[42] UNITAID. UNITAID Strategy Review 2017-2021. Available at: https://unitaid.org/assets/Unitaid-2017-2021-Strategy-Review_Final-Report_Vol-2-Annexes.pdf

[43] Clinton Health Access Initiative. New high-quality antiretroviral therapy to be launched in South Africa, Kenya and over 90 low- and middle-income countries at reduced price. Available at: https://www.clintonhealthaccess.org/news/new-high-quality-antiretroviral-therapy-launched-south-africa-kenya-90-low-middle-income-countries-reduced-price/

[44] PR Newswire. Viatris Inc. Announces FDA Tentative Approval of a Pediatric Formulation of Dolutegravir (DTG) Under PEPFAR. Available at: https://www.prnewswire.com/news-releases/viatris-inc-announces-fda-tentative-approval-of-a-pediatric-formulation-of-dolutegravir-dtg-under-pepfar-301179208.html

[45] Clinton Health Access Initiative. Prioritizing pediatrics: accelerating access to optimal pediatric HIV treatment. Boston: CHAI; 2021 Dec. Available from: https://www.clintonhealthaccess.org/report/prioritizing-pediatrics/

[46] Medicines Patent Pool. MPP Impact Analysis: HIV (2012-2018). Available at: https://medicinespatentpool.org/uploads/2019/07/DTG-Impact-Analysis_June19.pdf

[47] World Health Organization. Update on the transition to dolutegravir-based antiretroviral therapy. 2022. Available at: https://www.who.int/publications-detail-redirect/9789240053335

[48] Thornton I, Wilson P, Gandhi G. “No Regrets” Purchasing in a pandemic: making the most of advance purchase agreements. Global Health. 2022;18(1):62. doi: 10.1186/s12992-022-00851-3 35715814 PMC9204686

[49] Sterling TR, Villarino ME, Borisov AS, Shang N, Gordin F, Bliven-Sizemore E, et al. Three months of rifapentine and isoniazid for latent tuberculosis infection. N Engl J Med. 2011;365(23):2155–66. doi: 10.1056/NEJMoa1104875 22150035

[50] Sterling TR, Scott NA, Miro JM, Calvet G, La Rosa A, Infante R, et al. Three months of weekly rifapentine and isoniazid for treatment of Mycobacterium tuberculosis infection in HIV-coinfected persons. AIDS. 2016;30(10):1607–15. doi: 10.1097/QAD.0000000000001098 27243774 PMC4899978

[51] Swindells S, Ramchandani R, Gupta A, Benson CA, Leon-Cruz J, Mwelase N, et al. One Month of Rifapentine plus Isoniazid to Prevent HIV-Related Tuberculosis. N Engl J Med. 2019;380(11):1001–11. doi: 10.1056/NEJMoa1806808 30865794 PMC6563914

[52] Unitaid. Partners announce reduced price for patient-friendly tuberculosis preventive treatments. Available at: https://unitaid.org/news-blog/tb-preventive-therapy/#en

[53] MedAccess. Improving availability of patient-friendly tuberculosis preventive therapy. Available at: https://medaccess.org/improving-availability-of-patient-friendly-tuberculosis-preventive-therapy/

[54] Unitaid. Partners announce reduced price for patient-friendly tuberculosis preventive treatments. Available at: https://unitaid.org/news-blog/tb-preventive-therapy/#en

[55] TAG. New Rifapentine Formulations at New Prices: No Excuses for Not Scaling-Up TB Preventive Treatment. Available at: https://www.treatmentactiongroup.org/statement/new-rifapentine-formulations-at-new-prices-no-excuses-for-not-scaling-up-tb-preventive-treatment/

[56] TB alliance applauds Macleods’ launch of a key component of WHO-recommended drug-resistant TB treatment regimen. TB Alliance. Available at: https://www.tballiance.org/news/tb-alliance-applauds-macleods-launch-key-component-who-recommended-drug-resistant-tb-treatment

[57] Conradie F, Diacon AH, Ngubane N, Howell P, Everitt D, Crook AM, et al. Treatment of Highly Drug-Resistant Pulmonary Tuberculosis. N Engl J Med. 2020;382(10):893–902. 32130813 10.1056/NEJMoa1901814 PMC6955640

[58] Access to Medicine Foundation. Viatris leads in strategies to expand access to TB treatment (pretomanid). Available at: https://accesstomedicinefoundation.org/resource/viatris-leads-in-strategies-to-expand-access-to-tb-treatment-pretomanid

[59] TB Alliance. Realizing our “Triple-A Mandate. https://www.tballiance.org.za/news/realizing-our-triple-a-mandate

[60] MedAccess, Viatris and TB Alliance. Price reduction paves the way for expanded access to highly effective multidrug-resistant tuberculosis treatment. Available at: https://medaccess.org/price-reduction-paves-the-way-for-expanded-access-to-highly-effective-multidrug-resistant-tuberculosis-treatment/

[61] TBCAB. TB Alliance applauds Macleods’ launch of a key component of WHO-recommended drug-resistant TB treatment regimen. https://www.tbonline.info/posts/2023/11/13/tb-alliance-applauds-macleods-launch-key-component/

[62] Malhame M, Baker E, Gandhi G, Jones A, Kalpaxis P, Iqbal R, et al. Shaping markets to benefit global health - A 15-year history and lessons learned from the pentavalent vaccine market. Vaccine X. 2019;2:100033. doi: 10.1016/j.jvacx.2019.100033 31384748 PMC6668221

[63] Osoro CB, Ochodo E, Kwambai TK, Otieno JA, Were L, Sagam CK, et al. Policy uptake and implementation of the RTS,S/AS01 malaria vaccine in sub-Saharan African countries: status 2 years following the WHO recommendation. BMJ Glob Health. 2024;9(4):e014719. doi: 10.1136/bmjgh-2023-014719 38688566 PMC11085798

[64] PATH. Developing the world’s first malaria vaccine (RTS,S). (2024). https://www.path.org/our-impact/case-studies/developing-the-worlds-first-malaria-vaccine-rtss/

[65] MedAccess. RTS,S malaria vaccine. (2024). https://medaccess.org/our-agreements/agreements/rtss-malaria-vaccine/

[66] World Health Organization. 2022. WHO Guidelines for malaria. https://www.nitag-resource.org/sites/default/files/2022-05/Full-evidence-report-on-the-rtss-as01-malaria-vaccine-2021.pdf

[67] World Health Organization. WHO recommends groundbreaking malaria vaccine for children at risk. World Health Organization. 2021. https://www.who.int/news/item/06-10-2021-who-recommends-groundbreaking-malaria-vaccine-for-children-at-risk

[68] New financing agreement boost for malaria vaccine. https://www.gavi.org/news/media-room/new-financing-agreement-boost-malaria-vaccine

[69] Gavi, WHO and UNICEF. 18 million doses of first-ever malaria vaccine allocated to 12 African countries for 2023–2025. Available at: https://www.who.int/news/item/05-07-2023-18-million-doses-of-first-ever-malaria-vaccine-allocated-to-12-african-countries-for-2023-2025--gavi--who-and-unicef

[70] Unitaid, Médecins S, Frontières. Hepatitis C: Simple and effective treatment models in low-resource settings. Geneva: Unitaid. 2017.

[71] Avihingsanon A. Simplifying hepatitis C treatment in resource-limited settings: real-world outcomes from community-based programs. J Int AIDS Soc. 2018;21:e25099.

[72] Umutesi G, Shumbusho F, Kateera F, Serumondo J, Kabahizi J, Musabeyezu E, et al. Rwanda launches a 5-year national hepatitis C elimination plan: A landmark in sub-Saharan Africa. J Hepatol. 2019;70(6):1043–5. doi: 10.1016/j.jhep.2019.03.011 30948269

[73] World Health Organization. Eliminating hepatitis - Rwanda. World Health Organization. 2023. https://www.afro.who.int/countries/rwanda/news/eliminating-hepatitis#:~:text=Rwanda

[74] UNICEF, WHO, IFRC and MSF announce the establishment of a global Ebola vaccine stockpile. https://www.who.int/news/item/12-01-2021-unicef-who-ifrc-and-msf-announce-the-establishment-of-a-global-ebola-vaccine-stockpile

[75] Wilson PA, Thornton I, Gandhi G. Public health emergency archetypes: a framework to guide efforts to ensure equitable access to medical countermeasures. BMJ Glob Health. 2023;8(5):e012436. doi: 10.1136/bmjgh-2023-012436 37253532 PMC10230934

[76] WHO. Ebola virus disease: Vaccines. Available at: https://www.who.int/news-room/questions-and-answers/item/ebola-vaccines

[77] WHO. Ebola vaccine stockpiles. Available at: https://www.who.int/groups/icg/ebola-virus-disease/ebola-stockpiles

[78] Anderson EM, Coller B-AG. Translational success of fundamental virology: a VSV-vectored Ebola vaccine. J Virol. 2024;98(3):e0162723. doi: 10.1128/jvi.01627-23 38305150 PMC10994820

[79] Merck Confirms Agreement with UNICEF to Establish the World’s First Global Ebola Vaccine Stockpile with ERVEBO (Ebola Zaire Vaccine, Live) - Merck.com.

[80] Williams T, Taylor R, Iwamoto M, Hida T, Gusovsky F. The role of medicine donations in the global programme for the elimination of lymphatic filariasis. Int Health. 2020;13(Suppl 1):S39–43. doi: 10.1093/inthealth/ihaa077 33349878 PMC7753159

[81] Morin S, Moak HB, Bubb-Humfryes O, von Drehle C, Lazarus JV, Burrone E. The economic and public health impact of intellectual property licensing of medicines for low-income and middle-income countries: a modelling study. Lancet Public Health. 2022;7(2):e169–76. doi: 10.1016/S2468-2667(21)00202-4 34710359 PMC8809901

[82] United Nations Children’s Fund (UNICEF). Extension and expansion of the Vaccine Independence Initiative and its revolving fund. E/ICEF/2025/P/L.7. New York: UNICEF; 2024. Available from: https://www.unicef.org/executiveboard/media/28231/file/2025-PL7-Extension-vaccine-independence-EN-ODS.pdf

[83] UNICEF. The Vaccine Independence Initiative. Available at: https://www.unicef.org/supply/vaccine-independence-initiative

[84] Global Fund Announces New Mechanism to Increase Access to More Effective Mosquito Nets to Prevent Malaria. Available at: https://www.theglobalfund.org/en/news/2023/2023-08-22-global-fund-announces-new-mechanism-increase-access-more-effective-mosquito-nets-prevent-malaria/

[85] Partnerships for African Vaccine Manufacturing (PAVM) Framework for Action. Africa CDC. Available at: https://africacdc.org/download/partnerships-for-african-vaccine-manufacturing-pavm-framework-for-action/

[86] Africa CDC. The change in African vaccine manufacturing landscape. Available at: https://africacdc.org/wp-content/uploads/2024/11/The-change-in-African-vaccine-manufacturing-landscape-.pdf

[87] Osei-Akoto I, Kizito A, Asante FA. The Affordable Medicines Facility–malaria: achievements, challenges, and lessons learned. BMJ Global Health. 2020;5(12):e003741. doi: 10.1136/bmjgh-2020-003741

[88] Alderson MR, Sethna V, Newhouse LC, Lamola S, Dhere R. Development strategy and lessons learned for a 10-valent pneumococcal conjugate vaccine (PNEUMOSIL®). Hum Vaccin Immunother. 2021;17(8):2670–7. doi: 10.1080/21645515.2021.1874219 33625961 PMC8475595

[89] The Federal Democratic Republic of Ethiopia. Ethiopian Pharmaceutical Supply Agency Pharmaceuticals Supply Transformation Plan II (PSTP II) 2020/21-2029/30. Available at: http://repository.iphce.org/handle/123456789/2944

[90] Results for Development. Institutionalizing Market Shaping Capacity in Ethiopia through a Novel Systemic Approach. Available from: https://r4d.org/projects/institutionalizing-marketshaping-capacity-in-ethiopia-through-a-novel-systemic-approach/

[91] Gavi. Partnering with multilateral development banks to build sustainable immunization systems. Available at: https://www.gavi.org/sites/default/files/white-paper/2025/background-paper-Multilateral-development-banks_ENG.pdf

[92] Malhame M, Baker E, Gandhi G, Jones A, Kalpaxis P, Iqbal R, et al. Shaping markets to benefit global health - A 15-year history and lessons learned from the pentavalent vaccine market. Vaccine X. 2019;2:100033. doi: 10.1016/j.jvacx.2019.100033 31384748 PMC6668221

[93] Mpabalwani EM, Sakala C, Kamiji E, Simwaka J, Soko J, Kabwe M, et al. Challenges and lessons learned during the switching of rotavirus vaccine from Rotarix to Rotavac in Zambia. Vaccine. 2025;55:127012. doi: 10.1016/j.vaccine.2025.127012 40107130

[94] World Health Organization. African Medicines Regulatory Harmonization (AMRH): Progress report 2023. Geneva: World Health Organization; 2023

[95] Beaulieu-Jones BK, Finlayson SG, Yuan W, Altman RB, Kohane IS, Prasad V, et al. Examining the Use of Real-World Evidence in the Regulatory Process. Clin Pharmacol Ther. 2020;107(4):843–52. doi: 10.1002/cpt.1658 31562770 PMC7093234

[96] Philpott-Morgan S, Thakrar DB, Symons J, Ray D, Ashrafian H, Darzi A. Characterising the nationwide burden and predictors of unkept outpatient appointments in the National Health Service in England: A cohort study using a machine learning approach. PLoS Med. 2021;18(10):e1003783. doi: 10.1371/journal.pmed.1003783 34637437 PMC8509877

